# Atypical Intracavitary Cardiac Mass: Tumor or Thrombus?

**DOI:** 10.7759/cureus.21937

**Published:** 2022-02-05

**Authors:** Adeyinka Adeniyi, Sandra Abadir, Kalindi Parikh, Radhika Khanna, Sunday Yusuf, Marie Anais Hichard

**Affiliations:** 1 Internal Medicine, Wellstar Atlanta Medical Center, Atlanta, USA; 2 Cardiology, Wellstar Atlanta Medical Center, Atlanta, USA; 3 Family Medicine, Wellstar Atlanta Medical Center, Atlanta, USA

**Keywords:** hospital cost, cardiac mri, cardiac ct, echo contrast perfusion imaging, tumor or thrombus, right ventricular cardiac mass

## Abstract

Cardiac masses are a rare finding, with most masses found being thrombi or vegetations. Still, some masses are suspected to be a tumor based on multiple characteristics such as size, location, mobility, and the tumor's hemodynamic effects. Cardiovascular magnetic resonance (CMR) and CT have been shown to differentiate a tumor from a thrombus based on tissue characteristics of the mass. Here we highlight the role of contrast perfusion imaging on echocardiography in identifying the malignant potential of a cardiac mass. This case report demonstrates the effectiveness of contrast imaging with a transesophageal echocardiogram in identifying the etiology of cardiac masses without the need of CMR or cardiac CT, which helps save between $100-1207 of hospital costs. Besides the cost-benefit, the use of non-invasive and easily accessible imaging like echocardiogram enables smaller facilities with limited resources to diagnose and hence further manage patients with cardiac masses.

## Introduction

Cardiac masses are, in many cases, incidental findings seen on echocardiograms ordered for other indications. It has been established that cardiac metastasis is 30 times more common than primary cardiac tumors [1]. Furthermore, most cardiac masses are more likely to be a thrombus or vegetation than malignant [2]. Once a cardiac mass has been discovered, further imaging is required to determine the etiology of the mass to assess further management. Characteristics of the mass such as the size, location, mobility, vascularity, and their solid or cystic nature can be useful in differentiating a tumor from a thrombus [3]. Cardiac magnetic resonance (CMR) has been shown to help achieve the differentiation between a tumor and thrombus based on the characteristics of the tissue. A retrospective study involving 116 cases in which a CMR was used to differentiate tumor from thrombus showed that a thrombus was consistently small, less mobile, and homogeneous in nature while large masses with irregular margins, broad-based attachments, and masses that transverse the cardiac chamber were especially concerning for malignancy [4]. Cardiac CT is an alternative imaging modality used to assess cardiac masses in patients with contraindications to CMR [3]. Positron emission tomography (PET) coupled with fluorodeoxyglucose (FDG) is another imaging modality that helps assess the metabolic uptake by the cardiac mass and hence differentiates between benign and malignant masses [5]. The risk of contrast-nephropathy, cost burden, and lack of availability limits the use of imaging modalities like CMR, CT, and PET-FDG. Echo contrast perfusion imaging (ECPI) can help identify the etiology of a cardiac mass and allow for further management with significantly lower cost and no radiation exposure. 

## Case presentation

**Figure 1:**
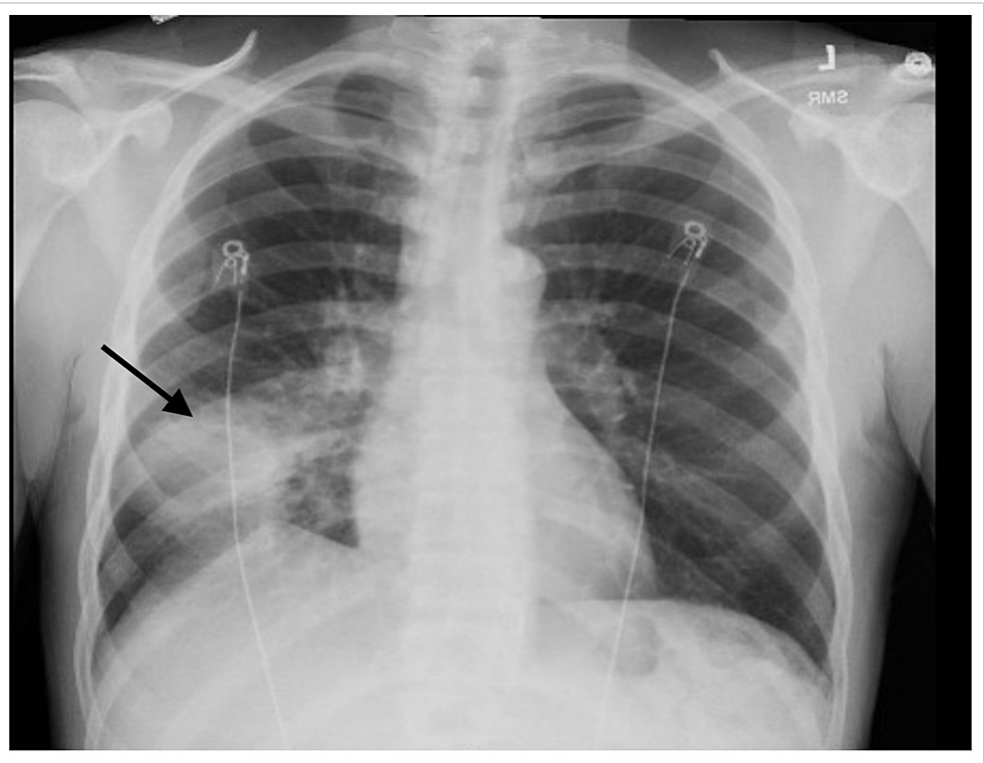
Posteroanterior chest X-ray on admission showing right lower lobe mass (arrow)

A 58-year-old male with a past medical history significant for newly diagnosed metastatic prostate cancer presented with a two-month history of progressive weakness, night sweats, dyspnea on exertion, hematuria, and a 40-pound weight loss. The patient denied headaches, vision changes, chest pain, palpitations, and shortness of breath at rest. He stated he was previously active until his recent cancer diagnosis and has never used illicit drugs. Vital signs were significant for fever, hypotension, and tachycardia. Physical examination showed a thin, fragile male with scleral icterus, periorbital edema, a diastolic murmur in the tricuspid region on cardiac auscultation, and a homogeneously enlarged prostate on digital rectal exam. Initial laboratory results revealed cardiac troponins 0.07 ng/mL x1 (peaked to 0.34 ng/mL x2), elevated WBC count of 29.8 10Eq/L, reduced hemoglobin of 7.3 g/dL, elevated total bilirubin of 8.5 mg/dL, alkaline phosphatase (ALP) of 253 IU/L, serum aspartate aminotransferase (AST) of 134 IU/L, and alanine transaminase (ALT) of 112 IU/L. His chest X-ray revealed a mass in the right lower lobe of the lung (Figure 1); CT of chest/abdomen/pelvis was significant for a mass in the right lower lobe of the lung with right hilar lymph node involvement. Furthermore, there was bony metastasis to T7 and T10 with a pathologic fracture to T10, left adrenal fullness, and an enlarged prostate gland with mass effect on the posterior urinary bladder. A CT-guided lung biopsy classified the mass as a poorly differentiated adenocarcinoma with replacement of normal lung parenchyma by malignant neoplasm and areas of fibrosis.

**Figure 2:**
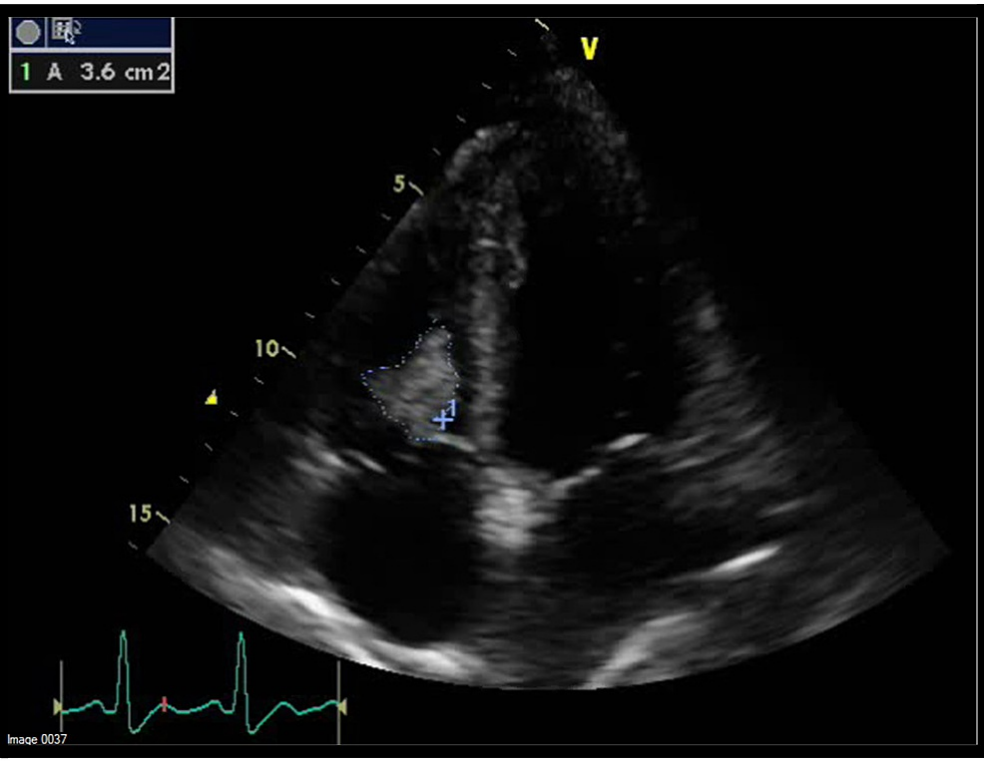
Transthoracic echocardiogram imaging with the echogenic mass in the right ventricle obstructing the tricuspid valve

**Figure 3:**
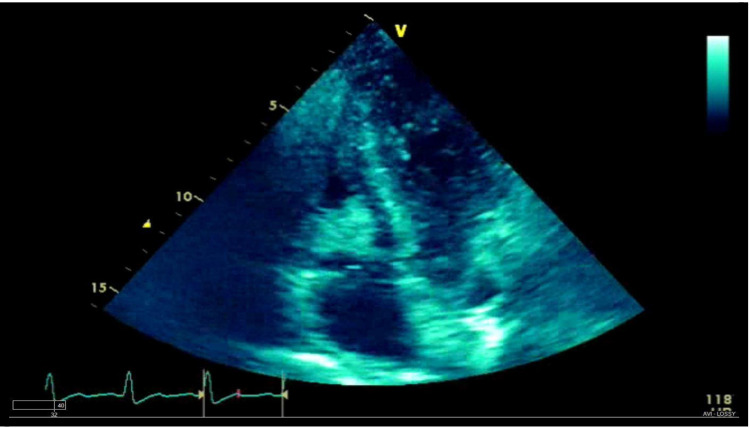
Transesophageal echocardiogram with Definity® contrast perfusion study showing contrast uptake by the right ventricle (RV) mass The mass in the right ventricle (RV) has irregular borders and shows significant contrast uptake.

A transthoracic echocardiogram (TTE) showed some pericardial effusion, a normal tricuspid valve structure with mild to moderate tricuspid valve regurgitation, and an echogenic mass obstructing the tricuspid valve. This echogenic mass was debated to be a tumor vs. thrombus considering the hypercoagulable malignant state the patient was in. The findings on TTE were most consistent with thrombus, but the possibility of a tumor could not be excluded (Figure 2). A transesophageal echocardiogram (TEE) performed two days later revealed a large, mobile, intracavitary mass in the right ventricle with significant uptake of echo contrast suggesting a tumor origin of the mass (Figure 3). 

Based on the patient's diffuse carcinomatosis and symptomatology, the patient was given a poor prognosis of only a few weeks. The patient opted for hospice care without any further intervention.

## Discussion

Most cardiac masses discovered on echocardiograms are thrombi or vegetation; these masses are rarely found to be tumors, and the ones that are malignant are more likely to be metastases than originate in the heart [2]. The clinical presentation of cardiac tumors depends on their size and location [6]. Most cardiac masses are asymptomatic and might go unnoticed until an autopsy; that being said, the most common clinical manifestation of these masses when symptomatic is hypotension, tachycardia, and shortness of breath [7]. 

Despite their rarity, cardiac masses, benign or malignant, need to be diagnosed and managed as they can be fatal. Identifying the location, size, mobility, myocardial invasion, and hemodynamic effect of the mass help determine the diagnosis and prognosis [1]. Echocardiography (sometimes ordered for other indications) is non-invasive, easily accessible, cheap, and lacks the need for contrast or radiation exposure, making it an ideal first-line imaging modality in diagnosing cardiac masses [2]. A three-dimensional TTE is favored over a two-dimensional TTE as it allows for full volume view, better resolution and reveals more information about the tumor location [1]. The addition of contrast to echocardiograms further helps in finding the etiology behind the cardiac mass by visualizing vascularity and the extent of contrast uptake [6]. 

Contrast echocardiography (CE) has been used widely in clinical cardiology for various clinical conditions [8]. An echocardiogram equipped with an S3 wideband transducer and contrast echo software is used to get images. After assessing the location, size, and mobility of the mass, a continuous infusion of contrast is delivered. The contrast used can be either a vial of Definity® (Lantheus Medical Imaging, Inc., North Billerica, USA) diluted in 60 ml saline solution and infused continuously or a 0.1 ml/kg of perfluorocarbon-exposed sonicated dextrose albumin (PESDA) diluted in 80 ml of 5% dextrose and given intravenously at a rate of 2-5 ml/min. The ultrasound beam is adjusted at the area of interest, and images are then acquired [9]. The contrast agents used in CE are microbubbles made up of a shell and encapsulated gas. The non-linear scattering of these contrast agents is what allows their detection by ultrasound. The microbubbles degree of swinging depends on how intense the ultrasound incident is [8]. This measure of intensity is called the mechanical index (MI). Once a mass demonstrates perfusion, an ultrasound impulse of high MI (>0.5) is transmitted to destroy the microbubble within the mass. The gas within the microbubble is then released to produce an acoustic signal which is detected by the ultrasound [8]. The signal reflected by the contrast agent indicates the concentration of the microbubbles within the myocardium. Once the myocardium is saturated with the microbubbles, the signal seen reflects the capillary blood volume [8]. Malignant masses demonstrate higher vascularity and, hence, higher contrast uptake compared to benign masses like thrombi that don't appear to enhance at all [6]. Our patient's TEE revealed significant echo contrast uptake on imaging, indicating high vascularization of the mass and hence malignant etiology. Further, the large size of the mass and its irregular borders makes it more likely malignant than thrombotic (as seen in Figures 2 and 3). 

While echocardiogram remains the first-line imaging modality, cardiac CT and CMR are other non-invasive modalities that have been shown to further characterize cardiac masses based on their solid or cystic nature, calcification, fat attenuation, and glucose metabolism. CMR is superior in visualizing the characteristics of cardiac masses in multiple plans, with high resolution and unrestricted views. However, the use of CMR is limited in patients that have implanted magnetic devices or are claustrophobic [3]. Cardiac CT range from $100-400 and exposes patients to more radiation, increasing the risk for contrast-induced nephropathy and further increasing the patient's hospital stay and cost of admission; CMR is even further expensive, ranging from $621 to $1,207. As discussed by Fathala et al. [5], PET has proven to be a valuable tool in evaluating cardiac masses. By utilizing FDG, it allows for an accurate assessment of metabolic activity of the cardiac masses hence differentiating between malignant and benign tumors. Malignant cardiac masses have high metabolic activity and, therefore, high FDG uptake. On the other hand, a thrombus is made up of activated platelets, macrophages, and fibrin with no metabolic activity and, therefore, no FDG uptake. Despite the valuable information provided by the PET-FDG, it is expensive and less available when compared to ECPI. However, one should keep in mind that in rare occasions, large chronic thrombi may enhance posing a diagnostic challenge [10]. In these cases, correlating the patient's presentation, symptoms timeline, and imaging is necessary.

## Conclusions

Patients with cardiac masses can stay asymptomatic or show symptoms of hemodynamic instability or dyspnea; these patients require an echocardiogram for initial diagnosis. Once the presence of a mass has been established, using echocardiogram contrast perfusion imaging (ECPI) helps differentiate between the etiology of the mass to further manage with chemotherapy vs. anticoagulation therapy vs. surgery vs. palliative care. Cardiac CT, CMR, and PET-FDG are other imaging modalities that can be utilized in finding the etiology behind cardiac masses. Although incredibly helpful, echocardiogram remains the first-line imaging modality of choice due to its wide availability, lack of contrast or radiation need, and low cost. Using ECPI alone could further reduce side effects from cardiac CT radiation exposure and lower overall hospital cost. The use of ECPI can also enable hospitals with a lack of advanced resources to establish the etiology of cardiac masses and further manage patients. 
